# The aggregation of cytochrome C may be linked to its flexibility during refolding

**DOI:** 10.1007/s13205-015-0345-y

**Published:** 2016-01-14

**Authors:** James I. Austerberry, Daniel J. Belton

**Affiliations:** 1 Manchester Institute of Biotechnology, University of Manchester, 121 Princess Street, Manchester, M1 7DN UK; 2 Department of Chemical Sciences, University of Huddersfield, Queensgate, Huddersfield, HD1 3DH UK

**Keywords:** Protein refolding, Molten globule, Fluorescence anisotropy, Protein aggregation, Protein flexibility

## Abstract

Large-scale expression of biopharmaceutical proteins in cellular hosts results in production of large insoluble mass aggregates. In order to generate functional product, these aggregates require further processing through refolding with denaturant, a process in itself that can result in aggregation. Using a model folding protein, cytochrome C, we show how an increase in final denaturant concentration decreases the propensity of the protein to aggregate during refolding. Using polarised fluorescence anisotropy, we show how reduced levels of aggregation can be achieved by increasing the period of time the protein remains flexible during refolding, mediated through dilution ratios. This highlights the relationship between the flexibility of a protein and its propensity to aggregate. We attribute this behaviour to the preferential urea-residue interaction, over self-association between molecules.

## Introduction

Biopharmaceutical production of therapeutic proteins including hormones and monoclonal antibodies (Shukla and Thömmes 2010) is a rapidly expanding area of research in biotechnology. However, the full benefits of this technology are yet to be fully realised as there are a number of barriers present in commercial production. One of these barriers is that of protein aggregation, where non-native protein monomers self-associate to form non-functional macromolecules; aggregates. The presence of these aggregates represents a loss of yield (and the cost of further processing in removing them), and their presence in therapeutics can trigger an immunogenic response, rendering the treatment ineffective (Sauerborn et al. 2010). Therefore, understanding the mechanisms of aggregation and development of protocols for reduction of aggregation are of high importance.

Post cellular production, quantities of the therapeutic protein take the form of inclusion bodies; a high concentration, insoluble mass aggregate of the recombinant protein that requires purification through refolding to obtain functional protein. High concentrations of denaturant (commonly urea) are required to solubilise the inclusion bodies and unfold the high-molecular weight aggregates (Basu et al. 2011). The preferential interaction (over that of water) amongst urea, the protein backbone and side chain groups are responsible for the unfolding of the aggregate at high denaturant concentrations (Canchi and García 2011; Guinn et al. 2011). These interactions drive the equilibrium of the protein towards the unfolded state with increasing concentration of denaturant (Canchi et al. 2010). Whilst in the presence of 8 M urea, it is possible that a protein still maintains some semblance of its native structure, forming a conformational space template for the protein to fold as limited hydrophobic interactions may still remain (Shortle and Ackerman 2001). Removal of the concentrated denaturant drives the collapse of the protein via hydrophobic and conformational backbone interactions (Haran 2012), wherein the protein folds to a low energy state. Whilst hydrophobic collapse drives the formation of the native state, the exposure of hydrophobic residues can lead to aggregation of protein monomers in high concentration environments (Fink 1998). Rapid refold dilution is common practice to instigate hydrophobic collapse and prevent hydrophobic exposure, however, Boismenu et al. (1997) showed that a slow refold dilution is capable of increasing refold yield significantly. Due to the time dependent nature of the effect, we postulate this may be attributed to mediation of late folding processes involved in residue rearrangement and structural mobility; the molten globule state, which occurs over a timescale of seconds (Arai and Kuwajima 1996).

Using multiple stopped flow techniques, it is possible to probe protein flexibility and investigate the role of final denaturant concentration in refolding in terms of aggregation. Fluorescence anisotropy is an analytical technique which offers the opportunity to probe the rotational mobility of fluorophores within the protein molecule, and provide information on their packing (Otto et al. 1994). It has previously been used to probe the mobility of chaperone complexes (Weissman et al. 1996), whilst experiments into refolding have clearly shown the existence of a molten state during refolding (Canet et al. 2001) but its use in relating this mobility to aggregation is novel. We combine this with stopped flow absorbance measurements to monitor aggregation during refolding. Here we present data for cytochrome C, a protein which contains no disulphide bonds, as a model system for biopharmaceuticals and show that the existence of its molten globule during folding is directly linked to its aggregation.

## Materials and methods

All chemicals were supplied by Sigma Aldrich, UK (reagent grade >99 % purity). Cytochrome C from bovine heart was provided as lyophilised powder from Sigma Aldrich, UK at >95 % purity as determined by SDS-PAGE. The protein was solubilised in 100 mM TRIS pH 10.6 buffer and unfolded in 8 M reagent grade urea solution. Concentration values of unfolded cytochrome C were produced at 1.6 × 10^−3^, 6.4 × 10^−4^ and 3.2 × 10^−4^ M as determined by ultra-violet spectroscopy, and sample absorbance at 320 nm was determined to be zero for each sample, indicative of no high-molecular weight aggregates being present from reconstitution (Murphy and Lee 2006). In stopped flow experiments, refolding of each sample of cytochrome C was initiated with dilution in 100 mM TRIS pH 10.6. This is achieved using a dual channel mixing system in the Applied Photophysics SX-20 in which a syringe containing the denatured protein and one containing the refold buffer are simultaneously driven to achieve a monodispersed mixing within a dead time of under 500 μs. The stopped flow fluorescence anisotropy and absorbance measurements were taken using the Applied Photophysics SX-20 stopped flow fluorescence anisotropy accessory. Hamilton syringes of 2.5 ml, 500, 250 and 100 µl were installed to provide mixing ratios 1:25, 1:10, and 1:5. Final concentrations for cytochrome C at each dilution was 6.4 × 10^−5^ M. Absorbance measurements were performed in a 1 cm pathlength orientation at 320 nm. Anisotropy measurements were performed by illuminating the sample at 280 nm based on the instrument wavelength selector coupled with a Schott UG-11 band pass filter on the inlet channel for excitation coupled with analysis of emitted light above 320 nm achieved with Schott WG-320 cut-off filters on the polarised output channels. Results presented are an average of at least 10 repeats.

The fluorescence anisotropy (*r*) is calculated using (1);

1 $$r = \frac{{I_{V} - I_{H} }}{{I_{V} + 2I_{H} }}$$

where the fluorescence anisotropy *r* is calculated from: *I*_*v*_, the fluorescence intensity of the vertically polarised emission and *I*_*H*_, the fluorescence intensity of the horizontally polarised emission. High values of *r* relate to a more stable fluorophores, and lower *r* values relate to fluorophores which are more mobile (Steiner 1991).

## Results and discussion

**Fig. 1:**
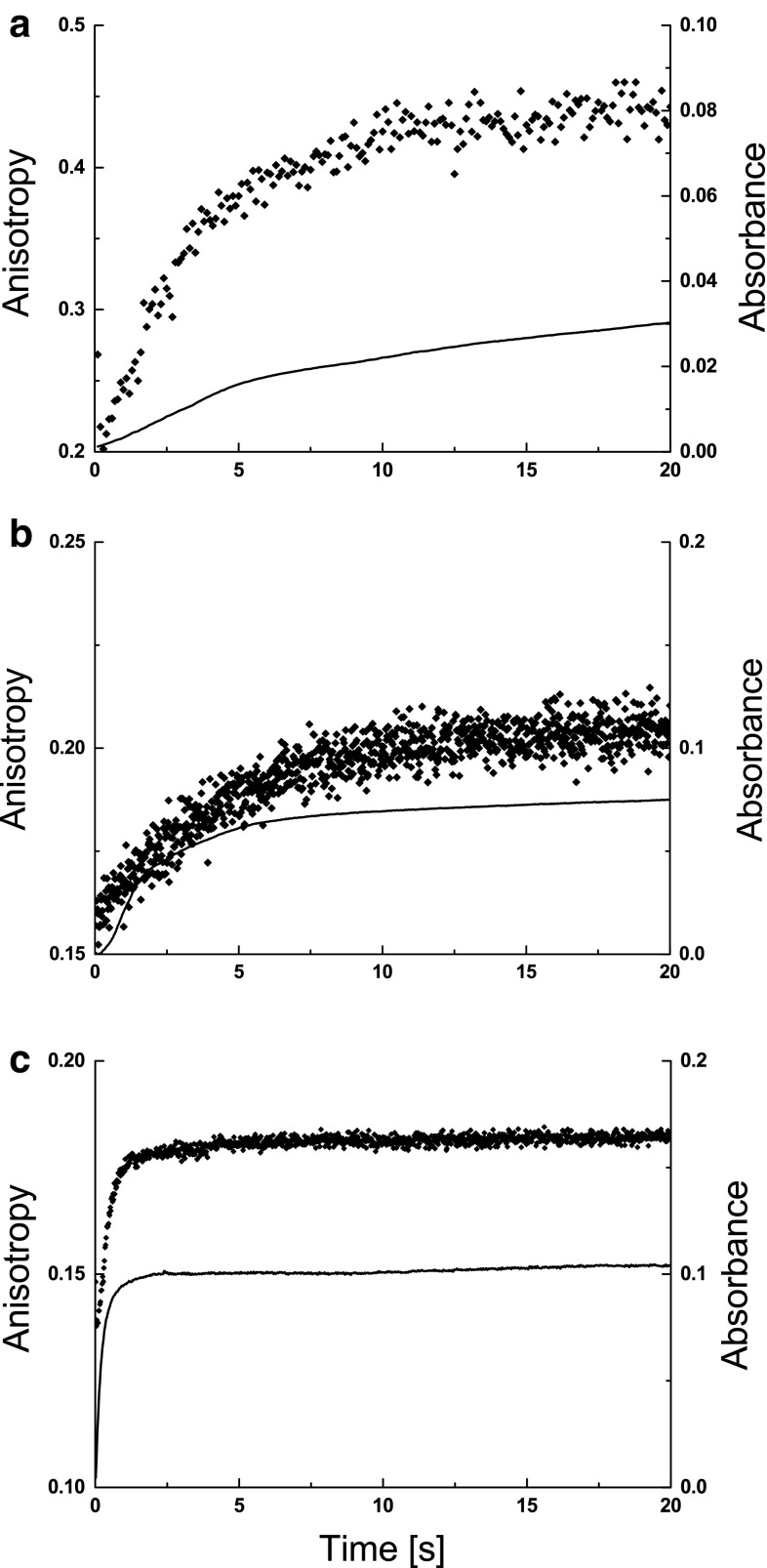
Fluorescence anisotropy at 280 (*diamond*) and absorbance at 320 nm (*solid line*) of cytochrome C in 8 M urea refolded at 1:5 (**a**), 1:10 (**b**) and 1:25 (**c**) by dilution with 100 mM TRIS pH 10.6, to a final protein concentration of 6.4 × 10^−5^ M

Refolding was initiated by the dilution of unfolded cytochrome C in 8 M urea with TRIS refolding buffer. To determine the effect of remaining urea concentration on the aggregation of cytochrome C, stopped flow refolding was performed at pH 10.6, marginally above the pI of the protein (Malmgren et al. 1978). This was chosen in order to prevent instability occurring from the solvation effects of the buffer, and to reduce long range charge–charge interaction effects at extremes of pH (Ibarra-Molero et al. 1999), in order to ensure that the contribution from unfolding is solely from the presence of the urea. Unfolding was undertaken at three ratios (1:5, 1:10, 1:25) (Fig. 1). The emergence of protein aggregates within the stopped flow sample can be measured by the absorbance of light at 320 nm (Murphy and Lee 2006), where scattered light from the aggregates manifests as an increase in intensity related to growth in size or frequency of the aggregate population.

The absorbance was observed to increase with time after the initiation of refolding. The final absorbance was greatest for the highest of the refold ratios (1:25), but lower when the refold ratio was decreased, where a 1:5 refold ratio results in a threefold decrease in absorbance over that of a 1:25 refold process (Fig. 1). As the mixing time occurs well within the dead time of this experiment, it is evident that aggregation is occurring in each of the dilution experiments, where aggregation is more prevalent at higher dilution ratios. The period over which the absorbance plateaus differs between refold ratios. At high refold ratio, the maximum value is achieved quickly, whilst at low refold ratios the aggregation takes place over a longer time period, taking circa five times longer to plateau. It is postulated that the protracted time period for this process is linked to the higher levels of urea concentration present in the final buffer (1.33 M urea for the 1:5 refold ratio and 0.73 M for the 1:10) versus that for the highest refold ratio (0.31 M for 1:25 refold ratio).

In order to probe the dynamics of this process, the mobility of the protein was studied through examination of the fluorescence anisotropy of cytochrome C at each refold ratio. The anisotropy was measured using excitation at 280 nm, here the signal is dominated by the emission from tryptophan residues. Bovine heart cytochrome C contains a single tryptophan residue at the base of one of the α-helices (2B4Z) (Berman et al. 2000). This residue, in combination with the 4 tyrosines, is buried in the hydrophobic core of the protein. Whilst the hydrophobic nature of the residues may lead to reduce mobility due to rapid hydrophobic collapse of the protein, any long term (>100 ms) mobility within this residue may give an indication of the timescale of side chain rearrangement in the latter stages of protein folding.

There is a marked difference in anisotropy signal produced at each refold ratio (Fig. 1). At low refold ratio, the change in anisotropy signal occurs over a 15-s time period, whilst at 1:25 the change occurs within circa 5 s. This indicates that at the lower refold ratios, cytochrome C remains in a more flexible conformation (molten globule state) than its native state for a longer period of time than at higher dilution ratios. This timescale appears to be well beyond the expected timescales attributed to hydrophobic collapse and alpha helices formation in cytochrome C (Akiyama et al. 2000, 2002), and in line with those expected from the molten globule (Arai and Kuwajima 1996).

Results presented here indicate there is a correlation between the flexibility of tryptophan side chain of cytochrome C during folding, and its propensity to form aggregates. At each refold ratio, the time period for side chain mobility to cease and absorbance to plateau are comparable. This change in anisotropy can be related to the existence of the molten globule intermediate. Whilst the protein is undergoing this transition in flexibility it is evident that aggregation is at its most prevalent. At increasing refold ratios the time period over which this transition takes place is reduced, a result which correlates with the remaining concentration of urea in the sample. Significantly, the increase in aggregation levels within the sample take place within during the same time period as the transition in flexibility, a result consistent for each refold ratio. This indicates a relationship between the flexibility within the protein, and its propensity to aggregate. Here, we postulate that the transition in flexibility is indicative of an aggregation prone intermediate in the aggregation pathway.

As the protein contains a single tryptophan, this residue may be responsible for the aggregation prone nature of the partially unfolded protein as a whole, or it may be indicative of the behaviour of other bulky hydrophobic side chains within the protein which more probably contribute to the aggregation of cytochrome C. The presence of urea sustains the existence of the molten state, but reduces the aggregation propensity of the molecule. We postulate that the preferential interactions between urea and the hydrophobic side chains prevent short-range attractive interactions that would lead to self-association between protein molecules and allow the hydrophobic residues to adopt an orientation buried from the surface. However, with lower the urea-side chain interactions present at higher refold ratios due to the decrease in relative urea concentration, the protein is forced to settle into a state where the energetics from hydrophobic exposure are instead reduced through association with a similarly perturbed aggregation prone molecule, resulting in aggregate formation.

These findings are consistent with refolding literature where the presence of moderate levels urea (1 M) will suppress the aggregation of papain in an acid induced molten globule state (Edwin et al. 2002) and that reducing refold ratio will suppress aggregation during refolding by dilution of recombinant protective antigen (Belton 2008). In this latter work, it was postulated that a short-lived molten globule intermediate was responsible for aggregation at higher refold ratios, but that reducing the refold ratio suppressed aggregation as a result of higher final urea concentrations. Here we have expanded upon these findings to highlight the time dependent correlation between protein flexibility and protein aggregation.

Overall, these findings indicate mediating the molten globule state existence appears critical in keeping aggregation levels low and thus maximising yield during the refolding from solubilised inclusion bodies. Whilst further work will examine the behaviour beyond the current model refolding system of cytochrome C, current results indicate that steps to elongate the existence of the molten globule, such as stepwise refolding, could be crucial to reducing aggregation during large-scale production of high value proteins.

## References

[CR1] Akiyama S, Takahashi S (2000). Stepwise formation of α-helices during cytochrome c folding. Nat Struct Mol Biol.

[CR2] Akiyama S, Takahashi S (2002). Conformational landscape of cytochrome c folding studied by microsecond-resolved small-angle X-ray scattering. Proc Natl Acad Sci.

[CR3] Arai M, Kuwajima K (1996). Rapid formation of a molten globule intermediate in refolding of α-lactalbumin. Fold Des.

[CR4] Basu A, Li X (2011). Refolding of proteins from inclusion bodies: rational design and recipes. Appl Microbiol Biotechnol.

[CR5] Belton D (2008) The physical characterisation of protective antigen protein. University of Manchester

[CR6] Berman HM, Westbrook J (2000). The protein data bank. Nucleic Acids Res.

[CR7] Boismenu R, Semeniuk D (1997). Purification and characterization of human and mouse recombinant alpha-fetoproteins expressed in *Escherichia coli*. Protein Expr Purif.

[CR8] Canchi Deepak R, García Angel E (2011). Backbone and side-chain contributions in protein denaturation by urea. Biophys J.

[CR9] Canchi DR, Paschek D (2010). Equilibrium study of protein denaturation by urea. J Am Chem Soc.

[CR10] Canet D, Doering K (2001). High-sensitivity fluorescence anisotropy detection of protein-folding events: application to α-lactalbumin. Biophys J.

[CR11] Edwin F, Sharma YV (2002). Stabilization of molten globule state of papain by urea. Biochem Biophys Res Commun.

[CR12] Fink AL (1998). Protein aggregation: folding aggregates, inclusion bodies and amyloid. Fold Des.

[CR13] Guinn EJ, Pegram LM (2011). Quantifying why urea is a protein denaturant, whereas glycine betaine is a protein stabilizer. Proc Natl Acad Sci.

[CR14] Haran G (2012). How, when and why proteins collapse: the relation to folding. Curr Opin Struct Biol.

[CR15] Ibarra-Molero B, Loladze VV (1999). Thermal versus guanidine-induced unfolding of ubiquitin. An analysis in terms of the contributions from charge-charge interactions to protein stability. Biochemistry.

[CR16] Malmgren L, Olsson Y (1978). Uptake and retrograde axonal transport of various exogenous macromolecules in normal and crushed hypoglossal nerves. Brain Res.

[CR17] Murphy RM, Lee CC (2006) Laser light scattering as an indispensible tool for probing protein aggregation. Misbehaving Proteins, Springer ebook

[CR18] Otto MR, Lillo MP (1994). Resolution of multiphasic reactions by the combination of fluorescence total-intensity and anisotropy stopped-flow kinetic experiments. Biophys J.

[CR19] Sauerborn M, Brinks V (2010). Immunological mechanism underlying the immune response to recombinant human protein therapeutics. Trends Pharmacol Sci.

[CR20] Shortle D, Ackerman MS (2001). Persistence of native-like topology in a denatured protein in 8 M urea. Science.

[CR21] Shukla AA, Thömmes J (2010). Recent advances in large-scale production of monoclonal antibodies and related proteins. Trends Biotechnol.

[CR22] Steiner RF (1991) Fluorescence anisotropy: theory and applications. Topics in fluorescence spectroscopy, vol 2. Plenum Press, J.R.Lakowicz. Baltimore

[CR23] Weissman JS, Rye HS (1996). Characterization of the active intermediate of a GroEL-GroES-mediated protein folding reaction. Cell.

